# The immune microenvironment after neoadjuvant therapy compared to upfront surgery in patients with pancreatic cancer

**DOI:** 10.1007/s00432-023-05219-7

**Published:** 2023-08-17

**Authors:** Eline S. Zwart, Thomas van Ee, Deesje Doppenberg, Arantza Farina, Johanna W. Wilmink, Eva Versteijne, Olivier R. Busch, Marc G. Besselink, Laura L. Meijer, Yvette van Kooyk, Reina E. Mebius, Geert Kazemier

**Affiliations:** 1 Department of Surgery, Amsterdam UMC, Vrije Universiteit Amsterdam, Amsterdam, The Netherlands; 2 Cancer Center Amsterdam, Amsterdam, The Netherlands; 3 Department of Molecular Biology and Immunology, Amsterdam Institute for Infection and Immunity, Amsterdam UMC, Vrije Universiteit Amsterdam, Amsterdam, The Netherlands; 4 Department of Surgery, Amsterdam UMC, University of Amsterdam, Amsterdam, The Netherlands; 5 Department of Pathology, Amsterdam UMC, University of Amsterdam, Amsterdam, The Netherlands; 6 Department of Oncology, Amsterdam UMC, University of Amsterdam, Amsterdam, The Netherlands; 7 Department of Radiation Oncology, Amsterdam UMC, Vrije Universiteit Amsterdam, Amsterdam, The Netherlands; 8 Oncology Graduate School, Amsterdam, The Netherlands

**Keywords:** Pancreatic cancer, Neoadjuvant therapy, Surgery, Tumor microenvironment, Immune system

## Abstract

**Background:**

Patients with resectable and borderline resectable pancreatic ductal adenocarcinoma increasingly receive neoadjuvant therapy prior to surgery. However, the effect of neoadjuvant therapy on the immune microenvironment remains largely unknown. We analyzed the immune microenvironment in pancreatic cancer tumor tissue samples from patients treated with neoadjuvant therapy compared to patients after upfront surgery to gain knowledge about the immunological environment after therapy.

**Methods:**

Multispectral imaging was performed on tissue from resected specimens from patients with PDAC who underwent upfront surgery (*n* = 10), neoadjuvant FOLFIRINOX (*n* = 10) or gemcitabine + radiotherapy (gem-RT) (*n* = 9) followed by surgery. The samples were selected by a dedicated pancreas pathologist from both the central part and the invasive front of the tumor (by the resected vein or venous surface) and subsequently analyzed using the Vectra Polaris.

**Results:**

Patients receiving neoadjuvant FOLFIRINOX display a more pro-inflammatory immune profile, with less regulatory T cells and more CD8 T cells in the tumor tissue compared to patients receiving neoadjuvant gem-RTgem-RT or undergoing upfront surgery. Furthermore, CD163^+^ macrophages were decreased, and a higher CD163^−^ macrophages versus CD163^+^ macrophages ratio was found in patients with neoadjuvant FOLFIRINOX. In all treatment groups, percentage of FoxP3^+^ B cells was significantly higher in tumor tissue compared to adjacent tissue. Furthermore, an increase in regulatory T cells in the tumor tissue was found in patients undergoing upfront surgery or receiving neoadjuvant gem-RT. In the gem-RT group, less CD8 T cells and a higher CD163^+^ macrophages to CD8 ratio were noted in the tumor tissue, suggesting a more immune suppressive profile in the tumor tissue.

**Conclusion:**

Patients receiving neoadjuvant FOLFIRINOX display a more pro-inflammatory immune profile compared to patients receiving neoadjuvant gem-RT or undergoing upfront surgery. Furthermore, in all treatment groups, a more immune suppressive microenvironment was found in the tumor tissue compared to the adjacent non-tumorous tissue.

**Supplementary Information:**

The online version contains supplementary material available at 10.1007/s00432-023-05219-7.

## Introduction

Patients with pancreatic ductal adenocarcinoma (PDAC) have the best survival if surgical resection of the tumor is feasible in combination with chemotherapy (Siegel et al. 2018). To increase overall survival (OS) rates, novel treatment strategies are currently investigated (Janssen et al. 2021; Anderson et al. 2021). Of particular interest is the application of neoadjuvant treatment (NAT), including chemotherapy with a combination of fluorouracil, leucovorine, irinotecan and oxaliplatin (FOLFIRINOX) or gemcitabine chemotherapy + radiotherapy (gem-RT) (Cloyd et al. 2020; Schorn et al. 2018; Zhan et al. 2017). Neoadjuvant treatment can reduce tumor size, improve radicality, decrease lymph node involvement (Youngwirth et al. 2017; Schorn et al. 2017; Roland et al. 2015; Ishikawa et al. 1994). However, little is known about the effect of NAT on the tumor microenvironment (TME) and specifically on the immune microenvironment (IM), although this appears to be of major influence on tumor response. Recently, few studies have been published describing a more pro-inflammatory immune profile after NAT (Dias Costa et al. 2022; Heiduk et al. 2022).

Neoadjuvant therapy drives the depletion of myeloid derived suppressor cells (MDSCs) and M2 macrophages by lowering growth factors and polarization of tumor associated macrophages into M1 macrophages via pro-inflammatory mediators (Mota Reyes et al. 2020; Michelakos et al. 2021; Caro et al. 2016). Furthermore, other studies have reported an increase in both CD4^+^ and CD8^+^ T cells, and a depletion of FoxP3^+^ T cells (Homma et al. 2014; Tsuchikawa et al. 2013). However, the differences between the effect of FOLFIRINOX and gem-RT on the immune microenvironment (IM) have not yet been compared for myeloid and lymphoid cells combined, while this could help to understand the effect of NAT.

For several other tumor types, immunotherapy has demonstrated tremendous response rates, resulting in increased OS and disease free survival (DFS) (Tumeh et al. 2014; Pawel et al. 2019; Herbst et al. 2014; Patnaik et al. 2015; Royal et al. 2010). However, for PDAC these response rates are not yet found. This is partly caused by the low mutational burden leading to less neopeptides compared to for example melanoma and by the immune suppressive environment of PDAC (Brouwer et al. 2022; Bailey et al. 2016; Balachandran et al. 2017; Balli et al. 2017). Understanding the effects of NAT on the immune microenvironment could help to decipher further the possible role of immunotherapy for patients with PDAC.

Therefore, to unravel the effect of NAT on the IM, we studied the IM after different treatment strategies consisting of upfront surgery, neoadjuvant FOLFIRINOX and neoadjuvant in patients with PDAC.

## Methods

### Study design

The study design and protocol were approved by the local Medical Ethical Board of the Amsterdam UMC in accordance with the ethical guidelines of the Declaration of Helsinki. Written informed consent was obtained from all participants before study participation (AMC2014_180; AMC2013_062; 2016.510). Clinical data were collected from the patient electronic file. The first available pre-treatment CA19-9 value, after biliary drainage if required, was selected as CA19-9 value. Preoperative tumor size was determined based on the first CT-scan and post-operative tumor size was based on the pathology report. Patients were operated on from 2010 to 2021. Therefore, the tumor stage was recalculated to the 8th edition of the American Joint Committee on Cancer (AJCC) classification for all samples based on the tumor size and positive lymph nodes (Chun et al. 2018).

In total, 4 μm formalin fixed paraffin embedded (FFPE) tissue slides from resection specimens of patients with PDAC after pancreatoduodenectomy, combined with a resection of part of the superior mesenteric or portal vein, or both and with available archival material, were included for immunohistochemical stainings. All patients, regardless of the treatment group, underwent a venous resection. Patients were divided into three groups, patients who underwent upfront surgery, patients treated neoadjuvant either with FOLFIRINOX or gem-RT. Patients were selected based on undergoing a venous resection as well as availability of paraffin embedded tissue. Of each patient, two slides were selected; one corresponding with the central part of the tumorbed and one corresponding with the invasive border at the periphery of the tumor, mainly at the venous surface or resected superior mesenteric/portal vein whenever venous invasion was present to determine spatial differences in immune composition. In both slides, ‘tumor’ and ‘tumor adjacent’ areas were indicated on a hematoxylin and eosin (H&E) slide by a dedicated pancreas pathologist (A.F.) (Supplementary Fig. 1).

### Immunohistochemistry

Slides were stained in accordance with the manufactures protocol with the Opal 7-color IHC kit (Akoya Biosciences, Catalog #NEL821001KT). In short, slides were deparaffinized and rehydrated in xylene and through graded concentrations of ethanol with MilliQ, followed by heat induced antigen retrieval buffer (Dako, Catalog #K800421-2) and cooling down to room temperature. Endogenous peroxidase was blocked using DAKO peroxidase block (Dako, Catalog #S202386-2). After thorough washes, sections were incubated with antibody diluent from kit (Akoya Biosciences, Catalog #NEL821001KT) to prevent aspecific binding, and subsequently incubated with a primary antibody. Slides were washed, incubated with secondary-HRP from the Opal 7-color IHC kit (Akoya Biosciences, Catalog #NEL821001KT), washed and incubated with Opal fluorophores or TSA-DIG from kit. Antibodies were removed using microwave treatment with antigen retrieval buffer and the protocol was repeated until all antibodies were stained. Slides were finally incubated with DAPI, washed, and mounted with Fluormount-G (ITK, Catalog #0100–01). The following primary antibodies were used: CD3 (Clone Sp7, Abcam, #ab86734), CD8 (Clone C8/144B, Dako Agilent, Catalog #M710301-2), FoxP3 (Clone 236A/E7, EBioscience, Catalog #14-4777-82), CD20 (Clone L26, ThermoFisher, Catalog #14-0202-82), CD68 (Clone PG-M1, Thermofisher, Catalog #MA5-12407), CD163 (Clone 10D6, ThermoFisher, Catalog # MA5-11458), Ki-67 (Clone MIB-1, Dako, Catalog #M724029-2), CD14 (Clone EPR3653, Abcam, Catalog #ab133335), PanCK (Clone AE1/AE3 + 5D3, Abcam, Catalog #ab86734), in combination with Opal Polaris 480 (Akoya Biosciences, Catalog #FP1500001KT), Opal Polaris 780 (Akoya Biosciences, Catalog #FP1501001KT) and Opal 520, Opal 540, Opal 570, Opal 620, Opal 650 and Opal 690 (Akoya Biosciences, Catalog #NEL821001KT). Slides were imaged using the Vectra Polaris.

### Image analysis

Acquired images were spectrally unmixed using inForm (version 2.6.0, Akoya Biosciences). Image analysis was performed using QuPath (version 0.2.2, Pete Bankhead). Cells were segmented using DAPI and the build-in watershed cell detection. Tumor and adjacent tissue annotations were copied from the annotated H&E slide. Immune and tumor cell phenotyping was done using Random Trees machine learning per individual marker and ultimately combined to a composite classifier. Immune populations were classified as the following: CD3^+^CD20^−^CD14^−^ Total T cells, CD3^+^CD8^+^FoxP3^−^ ‘cytotoxic T cells/CD8 T cells, CD3^+^CD8^−^FoxP3^+^ ‘regulatory T cells’, CD3^+^CD8^−^FoxP3^−^ ‘T helper cells / CD4 T cells’, CD20^+^CD3^−^ ‘total B cells’, CD20^+^FoxP3^+^ ‘FoxP3^+^ B cells’, CD3^−^CD20^−^CD14^+^ ‘myeloid cells’, CD14^+^CD68^−^CD163^−^ ‘monocytes’, CD14^+^CD68^+^CD163^−^ ‘CD163^−^ macrophages’, CD14^+^CD68^+^CD163^+^ ‘CD163^+^ macrophages’, and in the annotated tumor area CD3^−^CD20^−^CD14^−^PanCK^+^ ‘tumor cells’. Proliferation was based on Ki67^+^ signal for each cell type. Furthermore, the presence of tertiary lymphoid structures (TLS) was based on clustering of T and B cells.

### Statistical analysis

Clinical data were analyzed in SPSS (IBM SPSS Statistics version 26). For non-skewed continues data, the one-way ANOVA test was used and reported with the mean and standard deviation. In case of statistical differences between the groups, all groups were compared separately with a student’s *T* test. For skewed continues data, the Kruskal Wallis test was used and reported with the median and interquartile range. The Pearson’s Chi-squared test was used for ordinal and nominal data. For the correlation between categorical clinical data and immune subset abundances or ratio’s, the Kruskal Wallis test was used with a Bonferroni correction for multiple testing per clinical variable. For the correlation between continuous clinical data and immune subsets, the Pearson’s correlation test was used. For the survival analysis, abundances of immune populations were dichotomized based on the median abundance. Survival data were compared using Log rank test and presented with a Kaplan–Meier plot. A *p* value ≤ 0.05 was considered significant.

## Results

### Patients characteristics

Overall, 29 patients were included for this study, 10 patients in the upfront surgery group, 10 patients in the neoadjuvant FOLFIRINOX group and 9 patients in the neoadjuvant gem-RT treatment group. There were no differences between age and sex. Patients who received neoadjuvant FOLFIRINOX had a significantly larger tumor on preoperative imaging compared to patients who received neoadjuvant gem-RT. Noteworthy, in all patients having upfront surgery, margins were less than 1 mm in definitive pathology report, which was significantly more than patients receiving neoadjuvant FOLFIRINOX and neoadjuvant gem-RT (Table 1).

**Table 1:** Baseline characteristics

	Upfront surgery *N* = 10	Neoadjuvant FOLFIRINOX *N* = 10	Neoadjuvant gem-RT *N* = 9	*p* value
Age, years (SD)	62.3 (9.0)	67.5 (10.1)	69.0 (12.4)	0.36
Male sex, *n* (%)	5 (50.0)	5 (50.0)	5 (55.6))	0.96
	8.54 (0.9)	8.0 (1.0)	8.0 (0.7)	0.32
CA19.9 pretreatment, kU/L (IQR)	22 (78.5)	118 (114.5)	53 (332.5)	0.54
Biliary drainage, *n* (%)	7 (70.0)	4 (40.0)	6 (66.7)	0.33
Tumor size pretreatment, mm (IQR)	30.0 (6.0)	36.0 (16.8)	24.0 (11.0)	0.026^$^
ASA score, *n* (%)				
1	0 (0.0)	1 (10.0)	2 (22.2)	0.284
2	10 (100)	7 (70%)	6 (66.7)	
3	0 (0.0)	2 (20.0)	1 (11.1)	
Borderline resectable	5 (50.0)	9 (90.0)	5 (55.6)	0.128
Operation, *n* (%)				0.36
Pancreatoduodenectomy	10 (100.0)	7 (70.0)	8 (88.9)	
Distal pancreatectomy	0 (0.0)	2 (20.0)	1 (11.1)	
Total pancreatectomy	0 (0.0)	1 (10.0)	0 (0.0)	
Tumor size pathology, mm (IQR)	36.3 (8.8)	37.2 (16.9)	27.3 (6.1)	0.15
T stage*, *n* (%)				0.65
T2	7 (70.0)	5 (50.0)	5 (55.6))	
T3	3 (30.0)	5 (50.0)	4 (44.4)	
Lymph node involvement*, *n* (%)				
N0	4 (40.0)	5 (50.0)	8 (88.9)	0.17
N1	5 (50.0)	5 (50.0)	1 (11.1)	
N2	1 (10.0)	0 (0.0)	0 (0.0)	
R1 resection, *n* (%)	10 (100.0)	6 (60.0)	3 (33.3)	0.009^#^
Perineural invasion, *n* (%)	9 (90.0)	6 (60.0)	5 (62.5)	0.27
Angioinvasion, *n* (%)	2 (66.7)	3 (33.3)	3 (42.9)	0.598
Venous involvement	10 (100.0)	4 (40.0)	5 (55.6)	0.014^&^
Adjuvant therapy, *n* (%)	9 (90.0)	5 (50.0)	7 (77.8)	0.123

*According to the AJCC staging system, 8th edition

^$^Between neoadjuvant gem-RT vs neoadjuvant FOLFIRINOX, *p* = 0.034

^#^Between upfront surgery and neoadjuvant gem-RT *p* = 0.002 and between upfront surgery and neoadjuvant FOLFIRINOX *p* = 0.025

^&^Between upfront surgery and neoadjuvant gem-RT *p* = 0.0018 and between upfront surgery and neoadjuvant FOLFIRINOX *p* = 0.03

After staining and analysis, data of 28/29 slides were available on central tumor tissue and data of 24/29 slides were available on the invasive border. Unfortunately, during the staining process, some slides repeatedly lost adherence to the glass and could not be included for analysis. Finally, due to an exceptionally high autofluorescence in 4 cases, Ki67 signal could not be included for the proliferation analysis (Supplementary Fig. 2).

### Immune profile in central tumor and invasive border

First, we examined the immune subsets in general. For this, we included all samples per group, tissue from both the central tumor and from the invasive border at the periphery of the tumor at the resected vein or venous surface.

**Fig. 1:**
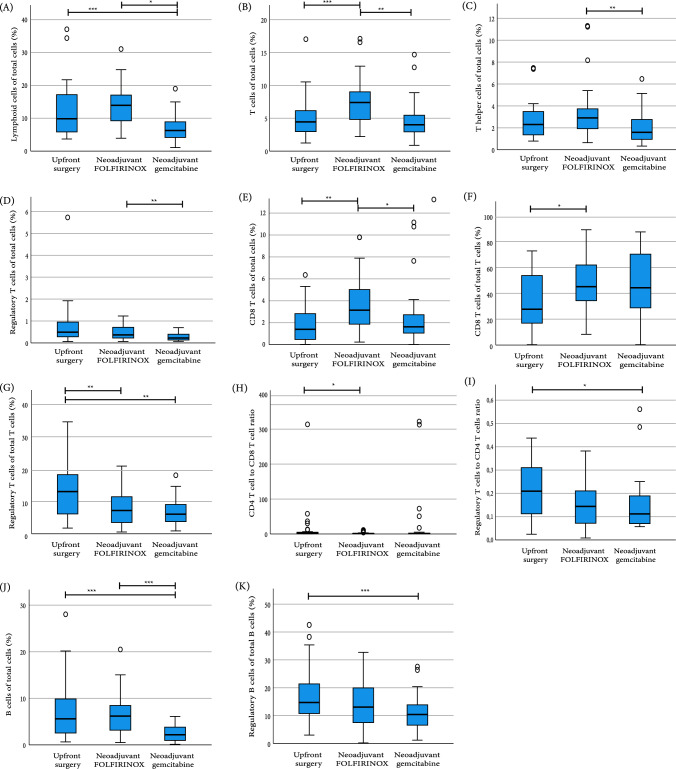
Abundance of lymphoid immune subsets and ratios between treatment groups. Abundance of **A** percentage of lymphoid cells of total cell population, **B** percentage T cells of total cell population, **C** percentage T helper cells of total cell population, **D** percentage of regulatory T cells of total cell population, **E** percentage of CD8 T cells of total cell population, **F** percentage of CD8 T cells of total T cell population, **G** percentage of regulatory T cells of total T cell population, **H** ratio of CD4 T cells to CD8 T cells, **I** ratio of regulatory T cells to CD4 T cells, **J** percentage of B cells of total cell population, **K** percentage of regulatory B cells of total B cell population. Boxplots: black bar denotes median, box denotes the interquartile range, whiskers indicate the range of values that are outside of the interquartile range. Outliers are defined as > 1.5 times the size of the interquartile range and presented as a circle. Statistical analysis performed with Kruskal Wallis test. *p ≤ 0.05 **p < 0.01, ***p < 0.001

There were significantly less lymphoid cells, consisting of CD3^+^ and CD20^+^ cells, in the gem-RT group compared to the upfront surgery and FOLFIRINOX group (Fig. 1A). Within the lymphoid subpopulations, there were significantly more T cells, based on expression of CD3^+^, in the FOLFIRINOX group compared to the gem-RT and upfront surgery group (Fig. 1B). This higher abundance appears to be caused by an increase in all T cells subsets, as there is an increase in T helper cells and regulatory T cells in the FOLFIRINOX group compared to the gem-RT group and an increase in CD8 T cells in the FOLFIRINOX group compared to the upfront surgery and gem-RT group (Fig. 1C–E).

Interestingly, when comparing the abundance within the T cells population, the CD8 T cells are significantly increased after FOLFIRINOX in comparison to upfront surgery and regulatory T cells are significantly decreased between FOLFIRINOX and upfront surgery group, but also between gem-RT and upfront surgery group (Fig. 1F, G). In addition, the ratio of CD8 T cells compared to CD4 T cells is higher in patients receiving FOLFIRINOX (Fig. 1H). Finally, patients receiving gem-RT had a lower regulatory T cell to CD4 T cell ratio, thus a smaller portion of the CD4 T cells are regulatory T cells compared to upfront surgery (Fig. 1I). These results demonstrate an increased abundance of lymphoid cells in patients treated with NAT, in particular, the ratios between the T cells appear to become more pro-inflammatory after NAT with FOLFIRINOX and to a lesser extend after gem-RT.

Furthermore, within the lymphoid subpopulations, there were significantly less B cells in the gem-RT group compared to the upfront surgery and neoadjuvant FOLFIRINOX group (Fig. 1J). In addition, among the B cells, there were significantly less FoxP3^+^ B cells in the gem-RT group compared to the upfront surgery group (Fig. 1K).

**Fig. 2:**
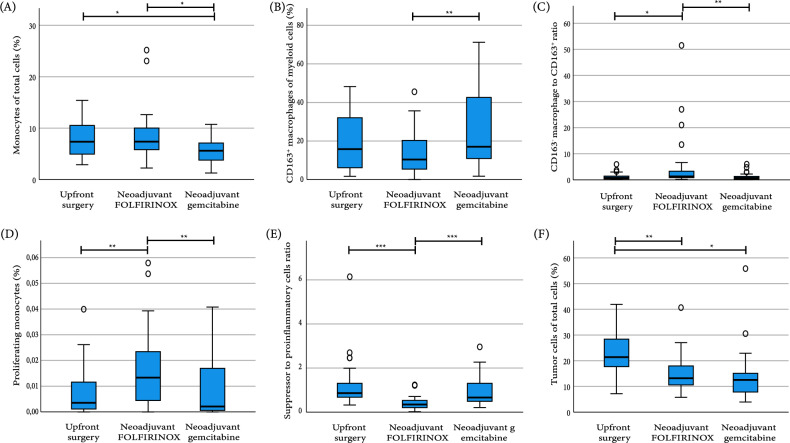
Abundance of immune subsets and ratios between treatment groups. Abundance of **A** percentage of monocytes of total cell population, **B** percentage of CD163^+^ macrophages of total myeloid cell population, **C** ratio of CD163^−^ macrophages to CD163^+^ macrophages, **D** percentage of proliferating monocytes of total monocytes population, **E** ratio of suppressor to pro-inflammatory cells, **F** percentage of tumor cells of total cell population. Boxplots: black bar denotes median, box denotes the interquartile range, whiskers indicate the range of values that are outside of the interquartile range. Outliers are defined as > 1.5 times the size of the interquartile range and presented as a circle. Statistical analysis performed with Kruskal Wallis test. *p ≤ 0.05, **p < 0.01, ***p < 0.001

In contrast to lymphoid cells, there were no differences in total abundance of myeloid cells between the three groups. However, within specific myeloid populations, there was a significant decrease in monocytes in the gem-RT group (Fig. 2A) and there was a higher percentage of proliferating monocytes after neoadjuvant FFX compared to upfront surgery and neoadjuvant gem-RT (Fig. 2D). Furthermore, within the myeloid cells, there was a significant decrease in CD163^+^ macrophages in the FOLFIRINOX group compared to the gem-RT group (Fig. 2B). The ratio between CD68^+^ and CD163^+^ macrophages was also higher in patients receiving neoadjuvant FOLFIRINOX, indicating that there are relatively more CD163^−^ macrophages to CD163^+^ macrophages compared to both the upfront surgery and gem-RT group (Fig. 2C).Thus, within the myeloid population, patients receiving neoadjuvant FOLFIRINOX had more activated monocytes and more CD163^−^ macrophages compared to CD163^+^ macrophages, once again suggesting a more pro-inflammatory immune profile compared to the immune profile of tumors after neoadjuvant gem-RT and upfront surgery.

Subsequently, the ratio between suppressive immune cells was examined, defined as regulatory T cells and CD163^+^ macrophages, and pro-inflammatory immune cells defined as cytotoxic T cells and CD163^−^ macrophages. Patients who received neoadjuvant FOLFIRNOX displayed the lowest ratio of suppressive pro-inflammatory immune cells compared to upfront surgery and gem-RT, indicating a more pro-inflammatory environment in these patients (Fig. 2E). Overall, patients receiving neoadjuvant FOLFIRINOX had a more pro-inflammatory immune profile compared to patients receiving gem-RT and patients undergoing upfront surgery.

Finally, percentage of tumor cells and proliferating tumor cells was studied. Patients receiving neoadjuvant gem-RT and FOLFIRINOX both had significantly lower amount of tumor cells compared to patients with upfront surgery (Fig. 2F), but there was no difference between gem-RT and FOLFIRINOX. Furthermore, there was no difference in the percentage of proliferating tumor cells between the three groups.

### Tumor versus adjacent tissue

First, tumor tissue was compared to the transitional border adjacent tissue on the same slide. In the upfront surgery group, there were significantly more myeloid and lymphoid cells in the adjacent tissue compared to the tumor tissue. This led to significant increases in B cells, T cells, T cell subsets including CD4 T cells, T helper cells, regulatory T cells and cytotoxic T cells and monocytes in the adjacent tissue of the upfront surgery group. Therefore, only percentages of parent immune populations (e.g., percentage of CD8 T cells of total T cells) or ratios were included for the comparison of tumor versus adjacent tissue.

**Fig. 3:**
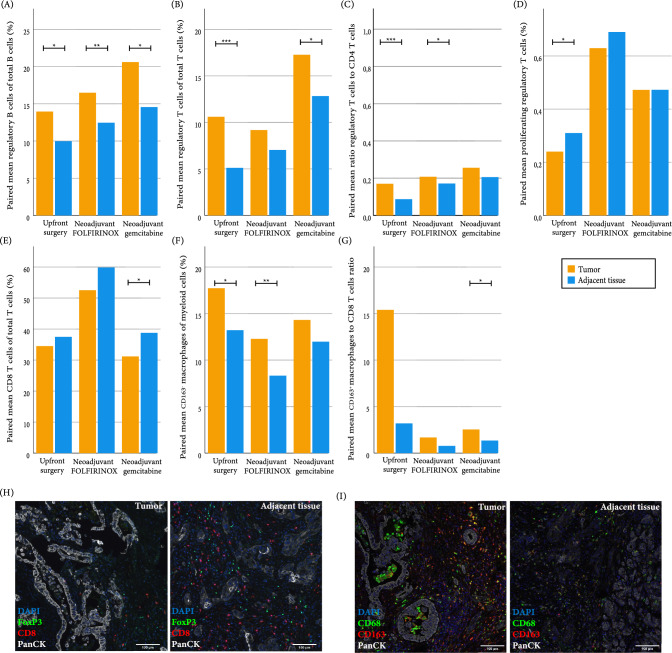
Paired analysis of immune subsets and ratios in tumor and adjacent tissue. Paired mean of **A** percentage of regulatory B cells of total B population, **B** percentage regulatory T cells of total T population, **C** ratio of regulatory T cells to CD4 T cells, **D** percentage of proliferating regulatory T cells of total regulatory T cells, **E** percentage of CD8 T cells of total T cell population, **F** percentage of CD163- macrophages of total myeloid population, **G** ratio of CD163^+^ macrophages to CD8 T cells, **H** representative image of differences between paired tumor and adjacent tissue for lymphoid cells (blue = DAPI, green = FoxP3, red = CD8, white = PanCK), **I** representative image of differences between paired tumor and adjacent tissue for myeloid cells (blue = DAPI, green = CD68, red = CD163, white = PanCK). Statistical analysis performed with paired *T* test. *p ≤ 0.05, **p < 0.01, ***p < 0.001

In all groups, there was a significantly higher percentage of FoxP3^+^ B cells of the total B cell population in the tumor tissue compared to the adjacent tissue (Fig. 3A). Furthermore, there was an increase in regulatory T cells of the total T cell population in the tumor tissue of patients undergoing upfront surgery and neoadjuvant gem-RT. A higher regulatory T cells to CD4 cells ratio was found in tumor tissue in upfront surgery and FOLFIRINOX patients. Also, in the upfront surgery group, a lower percentage of proliferating regulatory T cells was found in tumor tissue compared to the adjacent tissue (Fig. 3B–D). In the gem-RT group, there were more CD8 of the total T cells population in the tumor tissue (Fig. 3E). In the myeloid compartment, there were more CD163^−^ macrophages in the tumor tissue of upfront surgery and neoadjuvant FOLFIRINOX group (Fig. 3F). Finally, of the neoadjuvant gem-RT group, there was a higher ratio of CD163^+^ macrophages compared to CD8 cells in the tumor tissue, suggesting a more immune suppressive profile in the tumor tissue (Fig. 3G). Representative figures of the differences in paired samples are shown in Fig. 3H, I. In conclusion, a more immune suppressive profile was found in the tumor tissue compared to the adjacent tissue in the upfront surgery group, while after NAT, a mixed profile with FoxP3^+^ B and T cells, but also an increase of CD8 and CD163- macrophages was seen in the tumor tissue.

### Central tumor versus invasive front

**Fig. 4:**
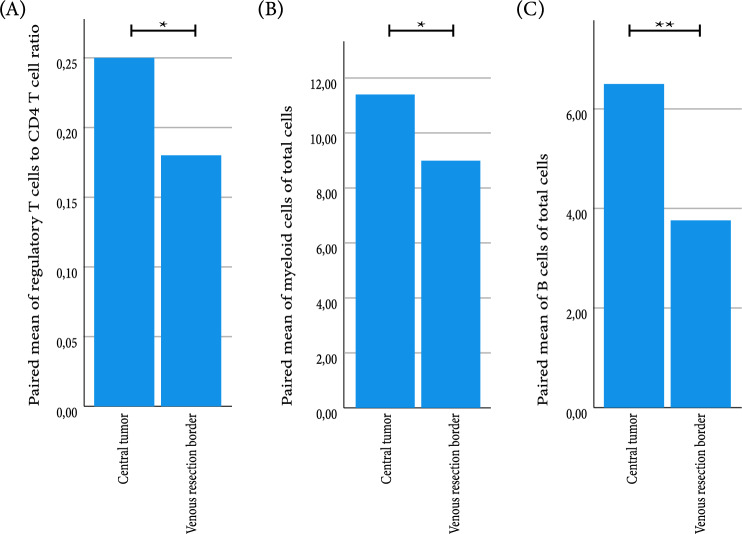
Paired analysis of immune subsets and ratios in tumor tissue at the central tumor and invasive border. Paired mean of **A** ratio of regulatory T cells to CD4 T cells (*n* = 21). **B** Percentage of myeloid cells of total cell population (*n* = 22). **C** Percentage of B cells of total cell population (*n* = 21). Statistical analysis performed with paired *T* test. *p ≤ 0.05, **p < 0.01, ***p < 0.001

To compare locations within a sample, we used a paired analysis of the central tumor tissue slide and tumor border. As there were many differences between tumor and adjacent areas, we separated the comparisons for these areas on the slide. First, we compared the tumor area of both locations and the adjacent area of both locations. Surprisingly, there were no differences between the immune subsets at the central tumor location and the invasive front in neither the upfront surgery group, FOLFIRINOX group and gem-RT group. When combining the three groups, there were significantly more regulatory T cells to CD4, more myeloid cells and more B cells in the tumor tissue of the central tumor slide compared to invasive front (Fig. 4A–C). There were no differences in the adjacent tissue between the central tumor and invasive front.

### Overall survival and disease-free survival

**Fig. 5:**
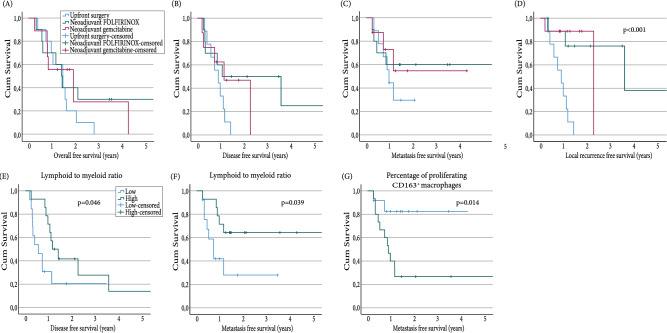
Overall and disease free survival analysis. Kaplan Meier survival curve for **A** overall survival for all treatment groups, **B** disease free survival for all treatment groups, **C** metastasis free survival for all treatment groups, **D** local recurrence free survival for all treatment groups, **E** disease free survival for high versus low lymphoid to myeloid ratio, **F** metastasis free survival for high versus low lymphoid to myeloid ratio, **G** local recurrence free survival for high versus low proliferating CD163^+^ macrophages. Statistical analysis performed with Log rank test

There was no difference in OS between the three groups (Fig. 5A). Next, we investigated whether the immune microenvironment correlated with survival. To this end, only the correlation between the immune subsets of the central tumor area was examined, but no correlation was found between the immune subsets and overall survival rates.

No differences were found in DFS or metastasis free survival between the treatment groups, although local recurrence free survival was significantly longer in patients treated with NAT (e.g., FOLFIRINOX or gem-RT-based (Fig. 5B–D). Next, survival outcomes for different immune subsets were compared. A longer DFS and metastatic free survival was found for patients with a higher lymphoid to myeloid ratio (Fig. 5E, F). Interestingly, less proliferating CD163^+^ macrophages correlated to a significantly increased metastastic free survival (Fig. 5G). The other immune subsets and ratios were not associated with DFS.

## Discussion

This study shows that the resection specimen of patients after neoadjuvant FOLFIRINOX displays a more pro-inflammatory immune microenvironment as compared to patients after neoadjuvant gem-RT based treatment and especially compared to patients undergoing upfront surgery. This is apparent from the lower ratio of immune suppressive cells (regulatory T cells and CD163^+^ macrophages) to pro-inflammatory cells (cytotoxic T cells and CD163^−^ macrophages). Furthermore, patients undergoing upfront surgery had a higher percentage tumor cells compared to patients who receive NAT in the examined slides.

To our knowledge, this is the first study that compares both the myeloid and lymphoid immune tumor microenvironment of patients receiving neoadjuvant gem-RT and neoadjuvant FOLFIRINOX to upfront surgery. Other studies have focused on either myeloid or lymphoid cells, or only one type of NAT (Dias Costa et al. 2022; Heiduk et al. 2022; Hwang et al. 2022). Furthermore, we examined different sites of the resected specimen, helping us understand the spatial differences. Apart from the more pro-inflammatory immune profile, we show that patients receiving neoadjuvant FOLFIRINOX have relatively more immune cell infiltration with more lymphoid cells in tumor tissue, with an increase in most T cell subsets, B cells, and more monocytes compared to patients receiving neoadjuvant gem-RT. Comparable to our results, Dias Costa et al. also showed a more pro-inflammatory immune profile after neoadjuvant FOLFIRINOX, with more CD8 and a higher CD8 to CD4 T cell ratio (Dias Costa et al. 2022). Furthermore, they also showed that in patients receiving neoadjuvant FOLFIRINOX, more macrophages and especially more CD163- macrophages were found. In their study, CD8 T cells and CD163- macrophages were more closely located near tumor cells after NAT (Dias Costa et al. 2022). Heiduk et al. also showed that after NAT, there was an increase in the total T cells population (Heiduk et al. 2022). Using flowcytometry, they also showed that after NAT, there are relatively less regulatory T cells of total CD4 T cell population. Furthermore, these CD4 T cells have a more pro-inflammatory profile, with an increase of TNF-a and less IL4. In addition, all T cells produce more IL-2 after NAT (Heiduk et al. 2022).

In a recent meta-analysis combining eight retrospective studies, NAT with FOLFIRINOX led to better OS compared to patients receiving neoadjuvant gemcitabine combined with NabPaclitaxel (Tang et al. 2021). It can be hypothesized that these favorable outcomes seen upon FOLFIRINOX treatment might be related to the higher abundance of pro-inflammatory immune cells, as these are able to attack tumor cells. This effect could be twofold: FOLFIRNOX might be more toxic to tumor cells, resulting in more tumor degradation. In addition, more tumor degradation results in more tumor derived material which can be presented by antigen presenting cells to lymphoid cells to start tumor specific adaptive immunity. Together, the combined effect of both chemotherapy and the immune system strengthens the overall effect of the therapy. In the study of Dias Costa et al., the increase of CD163^−^ macrophages and the location of CD163^−^ macrophages near the tumor cells, was also associated with a better response to neoadjuvant FOLFIRINOX and with a better survival (Dias Costa et al. 2022).

In the present study, more regulatory cells were found in the tumor tissue compared to the adjacent tissue in all three patient groups. This concerns both FoxP3^+^ B cells and regulatory T cells. Surprisingly, the proliferating regulatory T cells are more present in the adjacent tissue, suggesting they might be more active at the border or, as the total number of regulatory T cells is higher in the tumor tissue, that they migrate after development toward the tumor tissue. Mirlekar et al. also demonstrated more regulatory B cells in the tumor tissue compared to normal adjacent tissue (Mirlekar et al. 2022). Regulatory B cells produce immune suppressive cytokines such as IL-35 and IL-10 (Michaud et al. 2021). These can subsequently suppress CD8 T cells responses and enhance the proliferation of regulatory T cells (Mirlekar et al. 2020). In line with this, in our study the cytotoxic CD8 T cells were less prominently present in the tumor tissue compared to the adjacent tissue, suggesting a more pro-inflammatory immune system at the tumor border.

Compared to the invasive border at the periphery of the tumor at the resected vein or venous surface, a greater percentage of CD4 cells are regulatory T cells in the central tumor. It is thus expected that there is a gradient of the immune suppressive environment going from low at the outside border to high within the central tumor. Furthermore, there are also more myeloid and B cells in the central tumor. As there was no statistical difference found in the subsets of these cells, it is undetermined whether this constitutes of suppressive or pro-inflammatory cells. Brouwer et al. recently showed a higher abundance of myeloid and B cells in tumor tissue of patients with PDAC, but they compared it with matched normal tissue rather than with the invasive front/periphery of the tumor (Brouwer et al. 2022). They showed that the B cell population mostly consisted of naïve B cells followed by memory B cells. The role of B cells in PDAC remains controversial. In human studies, tumor infiltrating B cells are considered to be a positive prognostic factor, especially when B cells are located in tertiary lymphoid structures (Brunner et al. 2020; Castino et al. 2016; Tewari et al. 2013). However, in mice studies, B cells are associated with tumor progression (Mirlekar et al. 2020; Gunderson et al. 2016; Lee et al. 2016; Pylayeva-Gupta et al. 2016). In our study, we examined the presence TLS but did not find any significant difference between treatment groups, central versus invasive border or tumor versus adjacent tissue. Furthermore, we also did not find a significant correlation with survival. This might be due to the small study, however contradicting results regarding the effect on survival have been published (Gunderson et al. 2021; Delvecchio et al. 2021; Xuan et al. 2023).

For this study, only patients who underwent a venous resection were included. Patients who require a venous resection during surgery due to suspicion of contact between the tumor and the superior mesenteric or portal vein might have a more locally advanced tumor which is positively correlated with shorter OS (Giovinazzo et al. 2015). We deliberately choose to create a homogenous study group in which both NAT and upfront surgery are suggested in the current clinical guidelines, therefore only patients who underwent a venous resection were included (Bockhorn et al. 2014). Furthermore, the patients receiving NAT were treated within two randomized trials, which prevented selection bias in treatment allocation. However, in the baseline characteristics, there are still some differences. Patients receiving FOLFIRINOX had a larger tumor size at the start of treatment compared to patients receiving gem-RT. This might have had an influence on the capability of immune cells to penetrate to the tumor center. However, we did not find a difference in the percentage of tumor cells between patients receiving neoadjuvant FOLFIRINOX compared to gem-RT. Furthermore, in the upfront resection group, all patients had an R1 resection compared to 60% and 33% in the FOLFIRINOX and gem-RT based NAT groups, respectively. In the literature, an R1 resection is associated with worse OS and DFS. However, in our study, we did not find a difference in survival between both groups, possible due to a small sample size.

This study has several limitations. First, only small subsets of patients were included for this explorative study. This might have led to underpowered analysis in the comparisons between the immune subsets and the treatment groups, both in the abundances and survival analysis. For example in the comparison of the central tumor slide to the periphery of the tumor at the venous resection border, statistical differences where only found when all three subgroups were combined, possibly due to a higher sample size. Secondly, not all slides could be included for subsequent analysis due to damage during the antigen retrieval steps and were excluded from these analysis. However, the results of this explorative study could be the base for additional survival analyses on specific immune subsets in larger patient cohorts. Currently, it is unclear whether NAT, or adjuvant therapy, or a combination of both results in a better overall survival for patients with resectable PDAC (Versteijne et al. 2020). On the other hand, for borderline resectable PDAC, it has been shown that NAT leads to better OS compared to upfront surgery (Dam et al. 2022). Until recently, only adjuvant therapy was standard of care after surgical resection (Oettle et al. 2007). Novel insights have resulted in more aggressive therapeutic options, including NAT strategies resulting in enhanced survival rates compared to surgery only. In our study, 90% of patient undergoing upfront surgery also received adjuvant therapy, compared to 63% in the NAT group. Most patients of the upfront surgery group received gemcitabine based adjuvant therapy, as did the NAT gem-RT group. The neoadjuvant FOLFIRINOX group received mostly also FOLFIRINOX adjuvant therapy. This, together with the small sample size, could explain the similar survival rates in our patient groups, as other neoadjuvant studies did find a survival difference (Versteijne et al. 2022). Unfortunately, subgroup analyses on the immune subsets for each specific adjuvant treatment regimen could not be analyzed due to small numbers. Finally, only surgical material was used in this study. To study the direct effect of NAT, pretreatment tissue is required to study paired sample differences. Slides from patients undergoing upfront resection is now used as a substitute, but due to high interpatient heterogeneity might not completely resemble the effect of NAT. Therefore, further confirmative studies are required to compare the direct effect of NAT on the immune microenvironment.

The main strength of this study is that we compared whole slides analysis of the three most common treatment strategies currently used for (borderline) resectable PDAC. Furthermore, as PDAC is known for its high inter- and intratumoral heterogeneity, comparing different sites within one resected specimen helps us to better understand the differences between central tumor and peripheral invasive border. This can also help to understand the effect of NAT at different locations within the tumor, and possibly help steer targeted therapy to the desired location.

In conclusion, we show that patients after neoadjuvant FOLFIRINOX have a more pro-inflammatory immune microenvironment compared to patients after neoadjuvant gem-RT and patients who undergo upfront surgery. Furthermore, in the central tumor area the immune profile is more immune suppressive compared to the adjacent tissue in all treatment groups. These differences might help understand the effect of NAT on immune cells in patients with PDAC and could help to further investigate the role of immunotherapy for patients with PDAC. Ultimately, this could help in patient selection and optimize patient tailored therapy strategies.

## Supplementary Information

Below is the link to the electronic supplementary material.

Schematic overview of the selected slides and annotation Adjusted from Verbeke et al. (48) (PDF 1188 KB)

Flowchart of included samples after staining (PDF 380 KB)

## Data Availability

The datasets used and/or analyzed during the current study are available from the corresponding author on reasonable request.

## Abbreviations

PDAC: Pancreatic ductal adenocarcinoma
OS: Overall survival
NAT: Neoadjuvant treatment
FOLFIRINOX: Fluorouracil, leucovorine, irinotecan and oxaliplatin
TME: Tumor microenvironment
MDSCs: Myeloid derived suppressor cells
IM: Immune microenvironment
AJCC: American Joint Committee on Cancer
FFPE: Formalin fixed paraffin embedded
DFS: Disease free survival
