# Supplementary figures and images for: The immune microenvironment after neoadjuvant therapy compared to upfront surgery in patients with pancreatic cancer
Source: J Cancer Res Clin Oncol. 2023 Aug 17;149(16):14731–43. doi: 10.1007/s00432-023-05219-7 (PMC10603010; doi:10.1007/s00432-023-05219-7)

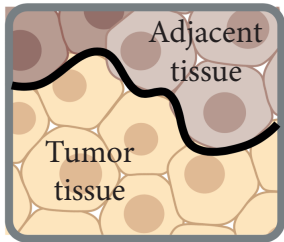

Central tumor

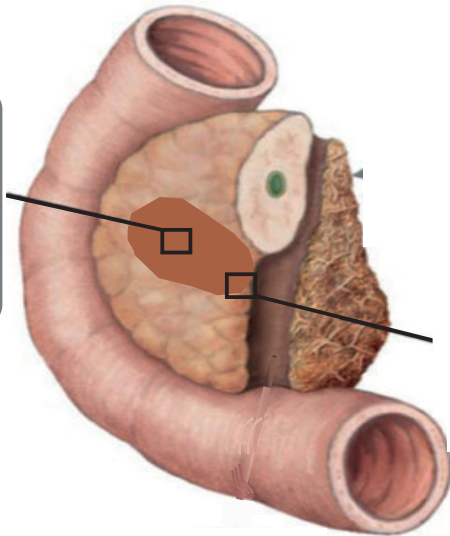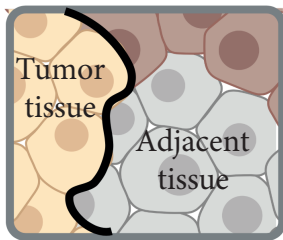

Venous resection margin

Supplement: Supplementary file 1 — Schematic overview of the selected slides and annotation Adjusted from Verbeke et al. (48) (PDF 1188 KB) [file 432_2023_5219_MOESM1_ESM.pdf]

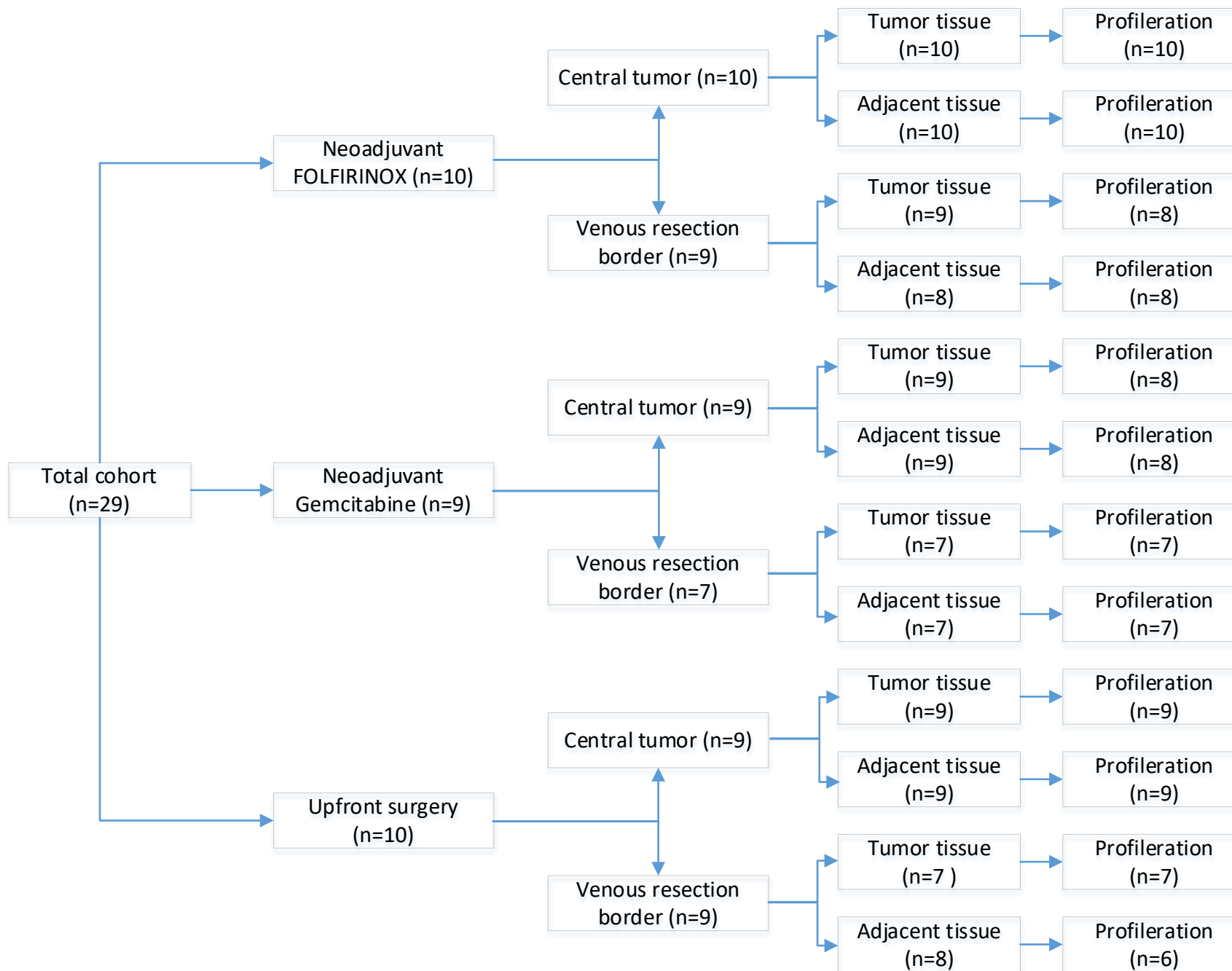

Supplement: Supplementary file 2 — Flowchart of included samples after staining (PDF 380 KB) [file 432_2023_5219_MOESM2_ESM.pdf]
